# Structure of sulfamidase provides insight into the molecular pathology of mucopolysaccharidosis IIIA

**DOI:** 10.1107/S1399004714002739

**Published:** 2014-04-30

**Authors:** Navdeep S. Sidhu, Kathrin Schreiber, Kevin Pröpper, Stefan Becker, Isabel Usón, George M. Sheldrick, Jutta Gärtner, Ralph Krätzner, Robert Steinfeld

**Affiliations:** a Department of Neuropediatrics, Faculty of Medicine, University of Göttingen, Robert-Koch-Strasse 40, 37075 Göttingen, Germany; b Department of Structural Chemistry, Institute of Inorganic Chemistry, University of Göttingen, Tammannstrasse 4, 37077 Göttingen, Germany; c Max Planck Institute for Biophysical Chemistry, Am Fassberg 11, 37077 Göttingen, Germany; d Instituto de Biologia Molecular de Barcelona (IBMB–CSIC), Barcelona Science Park, Baldiri Reixach 15, 08028 Barcelona, Spain; e Institucio Catalana de Recerca i Estudis Avancats (ICREA), Spain

**Keywords:** sulfamidase, mucopolysaccharidosis IIIA

## Abstract

Mucopolysaccharidosis type IIIA (Sanfilippo A syndrome), a fatal childhood-onset neurodegenerative disease with mild facial, visceral and skeletal abnormalities, is caused by an inherited deficiency of the enzyme N-sulfoglucosamine sulfohydrolase (SGSH; sulfamidase). More than 100 mutations in the SGSH gene have been found to reduce or eliminate its enzymatic activity. However, the molecular understanding of the effect of these mutations has been confined by a lack of structural data for this enzyme. Here, the crystal structure of glycosylated SGSH is presented at 2 Å resolution. Despite the low sequence identity between this unique N-sulfatase and the group of O-sulfatases, they share a similar overall fold and active-site architecture, including a catalytic formylglycine, a divalent metal-binding site and a sulfate-binding site. However, a highly conserved lysine in O-sulfatases is replaced in SGSH by an arginine (Arg282) that is positioned to bind the N-linked sulfate substrate. The structure also provides insight into the diverse effects of pathogenic mutations on SGSH function in mucopolysaccharidosis type IIIA and convincing evidence for the molecular consequences of many missense mutations. Further, the molecular characterization of SGSH mutations will lay the groundwork for the development of structure-based drug design for this devastating neurodegenerative disorder.

## Abstract 2

Mucopolysaccharidosis IIIA is a fatal neurodegenerative disease that typically manifests itself in childhood and is caused by mutations in the gene for the lysosomal enzyme sulfamidase. The first structure of this enzyme is presented, which provides insight into the molecular basis of disease-causing mutations, and the enzymatic mechanism is proposed.

## 1. Introduction   

Mucopolysaccharidoses (MPS) are a group of recessively inherited lysosomal storage disorders caused by a deficiency of enzymes involved in the metabolic breakdown of glycosaminoglycans (GAGs; also known as mucopolysaccharides). GAGs are unbranched polysaccharide chains consisting of repeating disaccharide units that contain variable modifications. In the cases of heparin and heparan sulfate, the degree of O- and N-sulfation is crucial for the biological function of these GAGs (Turnbull *et al.*, 2010 ▶).

At least 11 different enzyme deficiencies are currently known to be associated with the lysosomal storage of glyco­aminoglycans (Ballabio & Gieselmann, 2009 ▶). The inherited MPS disorders show multiple clinical symptoms such as facial dysmorphism, skeletal dysplasia, hepatosplenomegaly and variable signs of neurodegeneration (Clarke, 2008 ▶). In the subtypes III of mucopolysaccharidoses, also called Sanfilippo syndrome, signs of neurodegeneration are the initial symptoms and comprise of hyperactivity, developmental stagnation and psychomotor regression (Valstar *et al.*, 2010 ▶). Mucopolysaccharidosis type IIIA (MPS IIIA) is caused by the functional defect of N-sulfoglucosamine sulfohydrolase (SGSH; also known as sulfamidase, sulfamate sulfohydrolase and heparan N-sulfatase; EC 3.10.1.1) and represents one of the most frequent lysosomal storage diseases worldwide. Its incidence ranges from 0.68 per 100 000 to 1.21 per 100 000 in European countries (Héron *et al.*, 2011 ▶; Baehner *et al.*, 2005 ▶).

SGSH belongs to the sulfatase family and catalyses the cleavage of N-linked sulfate groups from the GAGs heparan sulfate and heparin. The active site is characterized by the amino-acid sequence motif C(*X*)PSR that is highly conserved among most sulfatases from all species. The leading cysteine is post-translationally converted to a formylglycine (FGly) residue, which is crucial for the catalytic process (Dierks *et al.*, 1999 ▶, 2005 ▶; Daniele & Di Natale, 2001 ▶).

Currently, there is no effective therapy available for MPS IIIA. Pathophysiological changes in the brain are the major determinants of the clinical picture. However, intravenous enzyme-replacement therapy is hampered by the very limited ability of the enzyme to cross the blood–brain barrier. While the structures of many O-sulfatases have been determined (Boltes *et al.*, 2001 ▶; Bond *et al.*, 1997 ▶; Hernandez-Guzman *et al.*, 2003 ▶; Lukatela *et al.*, 1998 ▶; Rivera-Colón *et al.*, 2012 ▶; van Loo *et al.*, 2010 ▶), no structures are available for an N-sulfatase such as SGSH. In addition, since SGSH shares only a very low sequence identity (approximately 19–25%) with O-sulfatases with known structures, understanding the molecular basis of this lethal disease is incomplete (Perkins *et al.*, 1999 ▶).

To understand the catalytic mechanism of human SGSH at the molecular level and to gain insight into the functional consequences of clinically relevant SGSH mutations, we determined its crystal structure in two crystal forms to resolutions of 2.00 and 2.40 Å. We compare the structure of SGSH as an enzyme cleaving N-linked sulfate groups with its closest O-sulfatase homologues that hydrolyze O-linked sulfates and discuss the structural consequences of clinically known mutations. Our results reveal structural deviations of SGSH from O-sulfatases and disclose unique features of its substrate-binding site.

## 2. Materials and methods   

### 2.1. Protein expression and purification   

The amino-acid sequence RSHHHHHH was introduced at the C-terminus of the SGSH protein to facilitate purification. Transfection of the modified SGSH cDNA and selection of HEK 293 cells were performed as described previously (Steinfeld *et al.*, 2004 ▶). Recombinant protein samples of SGSH were purified from the cell-culture supernatant. The medium was cleared by centrifugation at 3000*g* and 4°C for 60 min and was then filtered with a 0.2 µm pore membrane. After adding 20 m*M* K_2_HPO_4_ pH 7.5, 0.5 *M* NaCl, 40 m*M* imidazole, the crude solution was loaded onto a HisTrap HP column (GE Healthcare, Freiburg, Germany). Bound SGSH was eluted at an imidazole concentration of 250 m*M* with a step gradient. For crystallization, the purified SGSH fractions were pooled and concentrated using a centrifugal filter (Millipore, Schwalbach, Germany). The final protein solution contained 10 mg ml^−1^ SGSH and was adapted to 10 m*M* Tris pH 7.5, 100 m*M* NaCl. Enzyme purity was checked using SDS–PAGE.

### 2.2. Protein crystallization   

We obtained two different crystal forms of SGSH: a small-cell (S) form and a large-cell (L) form. Both forms were obtained using the sitting-drop vapour-diffusion method at 293 K. The S form was obtained in robotic crystallization screening trials set up in 96-well Greiner plates using Tecan Genesis RSP 150 and TTP Labtech Mosquito robots. The reservoir and drop volumes were 100 and 0.1 µl, respectively. The drop was prepared by mixing the protein solution with the reservoir solution in a 1:1 ratio. Rod-shaped crystals of maximum dimensions 20 × 20 × 50 µm grew over several weeks in 25%(*w*/*v*) polyethylene glycol (PEG) 3350, 200 m*M* MgCl_2_, 100 m*M* HEPES buffer pH 7.5. The L form was obtained during manual optimization trials, with rod-shaped crystals of typical dimensions 50 × 50 × 350 µm, in 13%(*w*/*v*) PEG 8000, 200 m*M* MgCl_2_, 100 m*M* bis-tris buffer pH 5.1.

### 2.3. Data collection   

Native single-crystal X-ray diffraction data were collected on the PXII beamline at the Swiss Light Source using monochromatic radiation of wavelength 0.99989 Å with a Pilatus 6M detector, an oscillation range of 0.1° and an exposure time of 0.1 s. A total of 3600 images were collected for the S crystal form, for which the space group was determined to be *P*2_1_, with unit-cell parameters *a* = 61.4, *b* = 107.9, *c* = 79.8 Å, β = 104.1°; 1800 images were collected for the L crystal form, which also crystallized in space group *P*2_1_, with an approximately four times larger unit cell: *a* = 103.0, *b* = 211.6, *c* = 108.4 Å, β = 102.7°. Data reduction was performed using the programs *XDS* (Kabsch, 2010 ▶) and *XPREP* (Sheldrick, 2012 ▶). Resolution limits for the small and large cells were set at 2.00 and 2.40 Å, respectively, based on self-correlation coefficients (Karplus & Diederichs, 2012 ▶) of approximately 65% for each. Table 1 ▶ lists the data-collection and refinement statistics.

### 2.4. Structure solution and refinement   

The crystal structure of *Pseudomonas aeruginosa* sulfatase (Boltes *et al.*, 2001 ▶; PDB entry 1hdh), which shares a structure-based sequence identity of approximately 22% with SGSH, was used as the basis from which to derive search-model fragments for molecular replacement (MR) in the small cell, as implemented in the program *ARCIMBOLDO* (Rodríguez *et al.*, 2009 ▶), and to combine the outcome to design an optimal MR search model. An initial rotation–translation solution with two incomplete monomers in the asymmetric unit was found that was clearly discriminated by MR figures of merit from all other solutions. However, attempts to refine this solution as such failed. Systematic attempts were made to improve the solution by disassembling parts of the model based on structure-based sequence alignments with close homologues, rigid-body refinement of rigid groups in *Phaser* (McCoy *et al.*, 2007 ▶) as part of the *CCP*4 suite of programs (Winn *et al.*, 2011 ▶), and low-resolution rigid-body and jelly-body refinement in *REFMAC* (Winn *et al.*, 2011 ▶; Murshudov *et al.*, 1997 ▶, 2011 ▶), gradually increasing the resolution. The program *SCWRL*4 (Krivov *et al.*, 2009 ▶) was initially used to model and test secondary-structure-based side-chain rotamers. The criteria monitored included the log-likelihood gain and the fit of the model to the electron density. A significantly incomplete but refinable model was finally obtained. For the *R*_free_ set for cross-validation of the structure models, 5% of all reflections were put aside randomly for the small cell; *SFTOOLS* (Winn *et al.*, 2011 ▶), *MTZ*2*HKL* (Grune, 2008 ▶) and *XPREP *were used to place aside, in 21 thin shells (2.45–48.9 Å), 8462 (4.8%) of all reflections for the large cell.

A partial structural model for the S crystal form was then used as a search model to solve the structure of the L form using *Phaser*. This latter cell contained eight molecules in the asymmetric unit. Density modification with NCS averaging over the small-cell and large-cell crystal forms could now be used with the program *DMMULTI* as part of *CCP*4 (Winn *et al.*, 2011 ▶; Cowtan, 1994 ▶), facilitating structure building in the S form. Manual building could subsequently be complemented by piecemeal corrections derived from automatic chain tracing using the program *Buccaneer* as part of *CCP*4 (Winn *et al.*, 2011 ▶; Cowtan, 2006 ▶). A completed model was then used to re-solve and refine the large-cell structure. Model building was peformed using *Coot* (Emsley *et al.*, 2010 ▶). Maximum-likelihood refinement of model coordinates against the working set data was performed using *REFMAC*5.5 with local NCS restraints. Water molecules were added automatically or manually in *Coot*. Waters were deleted manually if the refined density was weak, if the *B* factor refined to values exceeding 80 Å^2^ or if the waters were too close to neighbouring atoms or too distant from the protein. In the final refinement macrocycles, H atoms were added in riding positions and TLS parameters were refined (Murshudov *et al.*, 2011 ▶). The final *R*_free_ (Brünger, 1992 ▶) and *R*_work_ are 22.99 and 19.20%, respectively, for the small cell and 24.47 and 21.57%, respectively, for the large cell. SGSH monomer superposition r.m.s.d.s were calculated using *Indonesia* (Madsen *et al.*, 2005 ▶); those for the dimer were calculated using *LSQKAB* (Kabsch, 1976 ▶). Figures were drawn using *PyMOL* (Mura *et al.*, 2010 ▶) or an in-house program (NSS, unpublished work). Accessible surface area was calculated using the *PISA* server (Krissinel & Henrick, 2007 ▶). Normalized accessible surface area (NASA) per atom per residue was calculated by setting the accessible surface area per atom per residue for Lys490 as 100 and expressing the values for other residues in relation to this as a percentage. A model of the substrate was docked using the program *AutoDock Vina* (Trott & Olson, 2010 ▶).

### 2.5. SGSH activity assay   

SGSH activities were determined by a fluorometric assay as described previously (Karpova *et al.*, 1996 ▶). The enzymatic activity of SGSH was measured in a two-step reaction: 4-methylumbelliferyl-α-d-*N*-sulfoglucosaminide (MU-αGlcNS) is desulfated by SGSH to become a substrate for α-gluco­sidase, which converts 4-methylumbelliferyl-α-d-*N*-sulfoglucos­amine (MU-αGlc) to methylumbelliferone (MU), which is a fluorescent compound. The amount of MU can be quantified at wavelengths of 360 nm (excitation) and 460 nm (emission) using an external MU standard curve. Briefly, SGSH from a stock solution (0.8 mg ml^−1^) was diluted 1:100 with water containing NaCl (0.9%) and BSA (0.2%). 10 µl of this SGSH solution was gently mixed with 20 µl substrate buffer (14.3 m*M* sodium barbital, 14.3 m*M* sodium acetate pH 6.5, 0.7% NaCl, 2.28 mg ml^−1^ MU-αGlcN) and 10 µl inhibitor solution, which was prepared using KH_2_PO_4_ or Na_2_SO_4_ in water to reach final concentrations of 500, 250, 100, 50, 10, 5, 2.5, 1 or 0.5 m*M* SO_4_^2−^ or PO_4_^3−^ in the mixture. As a control for full enzymatic activity, 10 µl pure water was added instead of the inhibitor solution. The reaction mixture was incubated for 2 h in a 96-well plate at 37°C and shaken at 300 rev min^−1^. Subsequently, 6 µl P_i_/Ci buffer (0.4 *M* sodium phosphate, 0.2 *M* citrate pH 6.7) and 10 µl α-glucosidase (10 U ml^−1^, 0.2% BSA) were added. After incubation for 24 h at 37°C, 150 µl stop buffer (0.175 *M* glycine/Na_2_CO_3_) was added, followed by fluorometric measurement of enzymatic activity.

## 3. Results   

Full-length human SGSH was expressed in HEK 293 cells and was purified from the cell-culture supernatant. A two-residue C-terminal linker followed by a six-His tag (residues 503–510) was used to assist in purification of the recombinantly produced enzyme.

Two crystal forms of glycosylated SGSH were grown at different pH values. The crystal form grown at pH 7.5 is designated crystal form S (small unit cell). The structure of this form was refined to 2.00 Å resolution. The form grown at pH 5.1 is designated crystal form L (large unit cell). Its structure was refined to 2.40 Å resolution. There are two molecules in the asymmetric unit of crystal form S and eight molecules in that of form L. The enzyme appears to exist as a homodimer in both crystal forms.

### 3.1. Model quality   

For crystal form S, continuous electron density of good quality was observed for both molecules in the asymmetric unit; residues 22–504 were modelled in chain *A* and residues 22–503 in chain *B*. For crystal form L, the quality of the electron density varied significantly for the eight molecules in the asymmetric unit, with better density for approximately half of the molecules (chains *A*–*D*); the number of modelled residues ranged from 485 (residues 21–505) in chain *A* to 481 (residues 22–502 with residues 185–186 unmodelled) in chain *F*. Since the protein ϕ/ψ angles were not restrained during refinement, they serve as an indicator of model quality. The Ramachandran plot (Lovell *et al.*, 2003 ▶; Ramakrishnan & Ramachandran, 1965 ▶) values and data-collection and refinement statistics are listed in Table 1 ▶. Asp94 is an outlier in all ten chains in the two crystal forms, but lies in good density.

### 3.2. Structure of the SGSH monomer   

**Figure 1:**
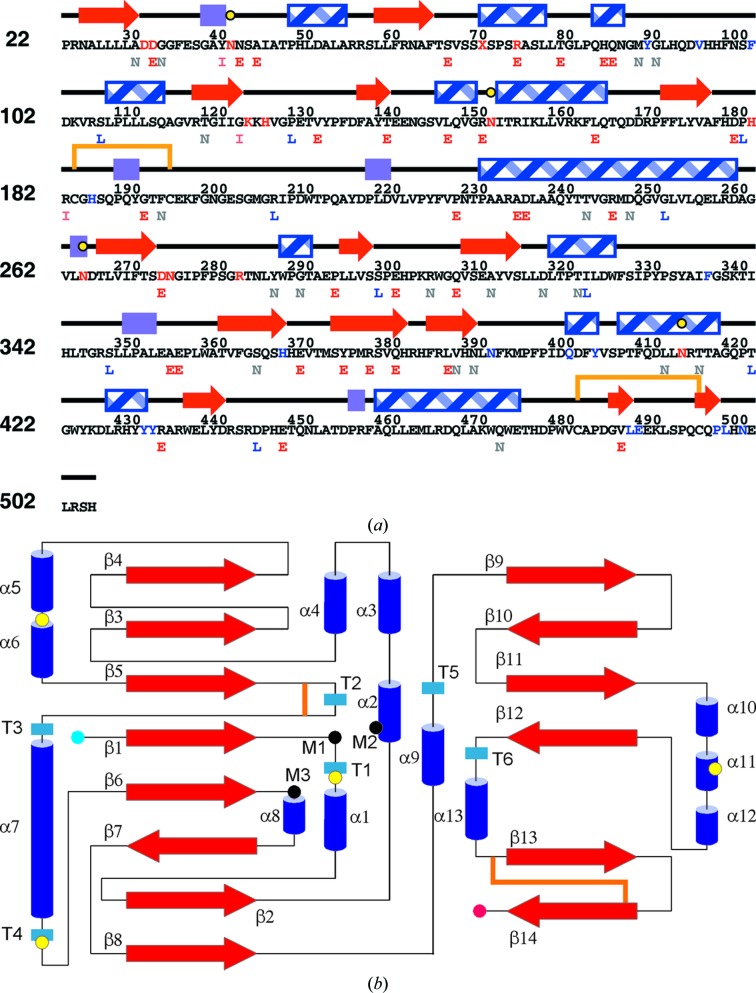
Schematic representations of the SGSH structure. (*a*) Mapping of SGSH primary and secondary structures. β-Strands, red arrows; α-helices, blue striped rectangles; 3_10_-helices, violet rectangles; the two disulfide bridges are shown as orange lines and the four glycosylated asparagines as yellow filled circles. Functionally important residues (active site and glycosylation sites) are shown in red; some of the residues at the dimer interface are shown in blue. The phenotype of the missense mutation sites is indicated below the sequence as follows: early-onset disease (E), red; intermediate-onset (I), orange; late-onset (L), blue; phenotype not reported in the literature (N), grey. (*b*) Topology diagram (not drawn to scale). Colour coding is similar to that in Fig. 1 ▶(*a*), with α-helices shown as blue cylinders and the N-terminus and C-terminus as blue and red circles, respectively. Divalent metal-binding residues are labelled M1 (Asp31, Asp32), M2 (FGly70) and M3 (Asp273, Asn274). Secondary-structure elements are named as indicated in the main text.

**Figure 2:**
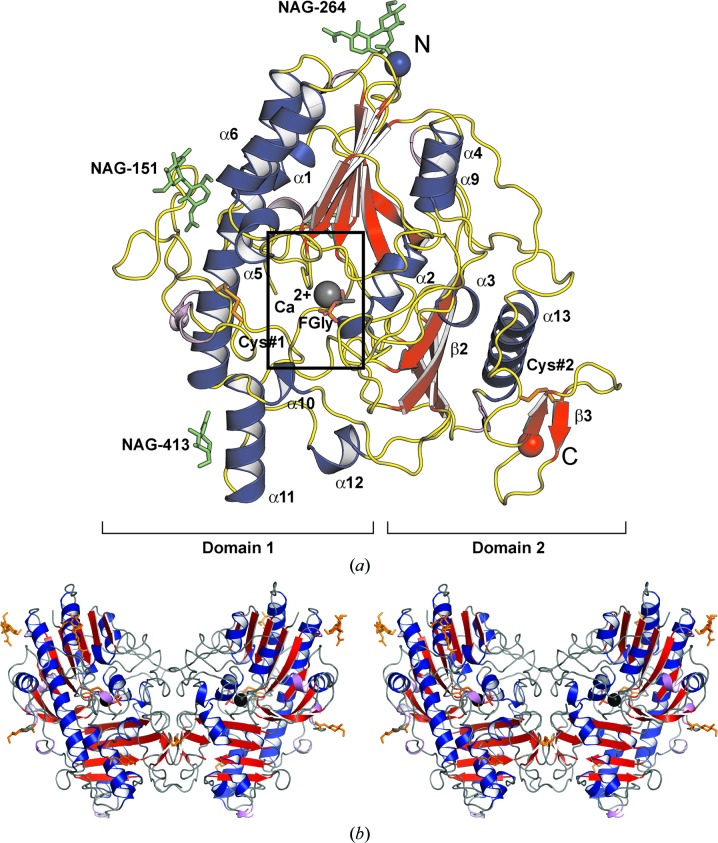

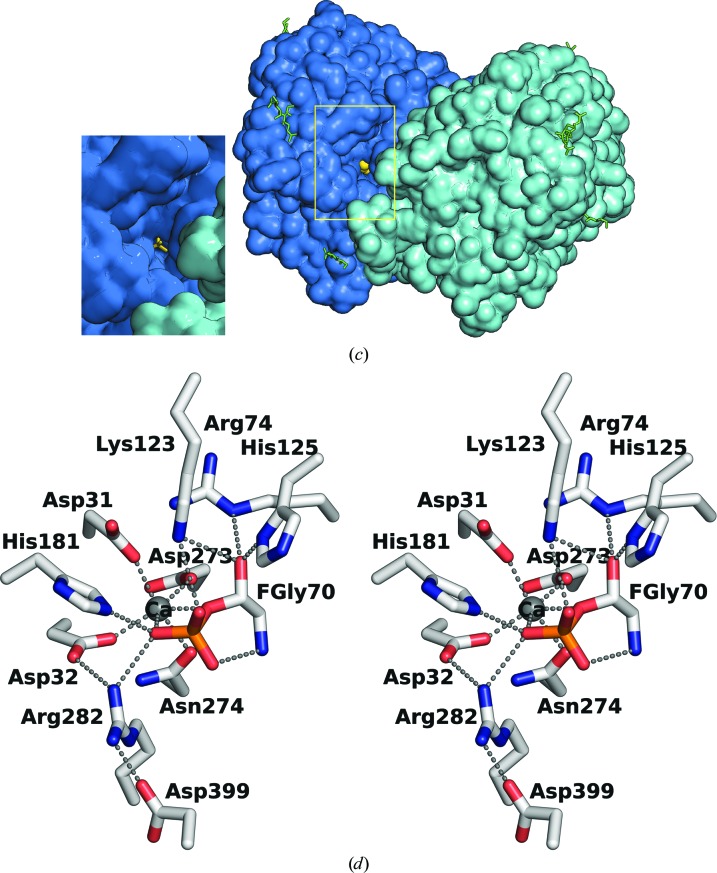
Three-dimensional structure of SGSH. (*a*) Monomer. The approximate locations of domains 1 and 2 are shown (square brackets), with β-sheets in domain 2 labelled β2 and β3. β-Strands are shown in red, α-helices in blue and loops in yellow. Cystine bridges are shown in orange (Cys#1, 183–194; Cys#2, 481–495). The N-terminus (N) is shown as a blue ball and the C-terminus (C) as an orange ball. The formylglycine (FGly) 70 side chain is shown as a stick model in standard colours. The Ca^2+^ ion is shown as a grey ball. Glycosylation sites (‘NAG-’ followed by the asparagine residue number) are shown as green sticks. (*b*) Dimer. The dimer noncrystallographic symmetry axis lies vertically in the plane of the paper, with subunit centroids in the approximate paper plane on either side of it. FGly70, cystine bridges and glycosylations are shown as orange stick models. Other representations are as in Figs. 1 ▶ and 2 ▶(*a*). (*c*) A short tunnel from a surface cleft leads to the active site. The inset on the left shows an enlargement of the boxed area. The two dimer subunits are shown in blue and cyan, FGly is shown as yellow spheres or sticks and glycosylations as green sticks. (*d*) Active site as viewed from its entry (stick models; the major interactions shown are described in the main text).

The crystal structure of glycosylated human SGSH was solved using molecular replacement. The monomeric enzyme subunit comprises of two domains, each centred on a β-sheet: a large N-terminal domain (domain 1) and a smaller C-terminal domain (domain 2), as is typical for the sulfatase fold. There are 14 β-strands, 13 α-helices and six 3_10_-helices (T1–T6) in total [Fig. 1 ▶; classification based on Kabsch & Sander (1983 ▶) as implemented in *PROCHECK* (Winn *et al.*, 2011 ▶)]. Domain 1 has an α/β form. Its core is formed by a mixed β-sheet consisting of eight β-strands, all except one of which are parallel, with nine decorating α-helices on both sides of the β-sheet (Fig. 1 ▶ *b*). One of the helices is 30 residues in length (helix α7). The core of domain 2 is formed by a four-stranded antiparallel β-sheet, with four surrounding α-helices, followed by a C-terminal extension consisting of a small two-stranded antiparallel β-sheet. The enzyme contains two intrasubunit disulfide bonds. One of these (Cys183–Cys194) stabilizes a long, loop-rich segment (β5–α7; residues 177–229) in domain 1. The second (Cys481–Cys495) ties the C-terminal extension to a proximal loop in domain 2 (Fig. 2 ▶ *a*). Electron density corresponding to glycosylated residues was observed at Asn41, Asn151, Asn264 and Asn413, in agreement with four of the five glycosylation sites previously reported (Di Natale *et al.*, 2001 ▶); no glycosylation could be detected at Asn142. There are four *cis*-peptide bonds in both chains, between prolines and the preceding Gly127, Asp179, Ala482 and Ser492; all of them lie in good density.

Two SGSH monomers associate noncovalently to form a ‘butterfly-shaped’ homodimer (Fig. 2 ▶ *b*), burying approximately 10.3% of the accessible surface area of each subunit.

### 3.3. SGSH shows low structural flexibility   

Since the SGSH structure was determined in two crystal forms, S and L, we could use the ten crystallographically independent monomers of SGSH to assess the structural flexibility of the enzyme under two different crystallization conditions. The enzyme subunits display a low rigid-body structural flexibility in the S form (C^α^ r.m.s.d. of 0.20 Å between the two chains, with NCS restraints) as well as the L form (r.m.s.d. ranging from 0.10 to 0.17 Å, with NCS restraints). To investigate the mobility at the dimer interface, all five homodimers were superimposed based on one of the subunits in each dimer. The relative orientation of the dimer subunits in the L form differs from that in the S form by 2.6–3.0°, indicating that the dimer interface is slightly flexible, possibly owing to crystal packing.

### 3.4. The active site and inhibition of SGSH by sulfate and phosphate   

**Figure 3:**
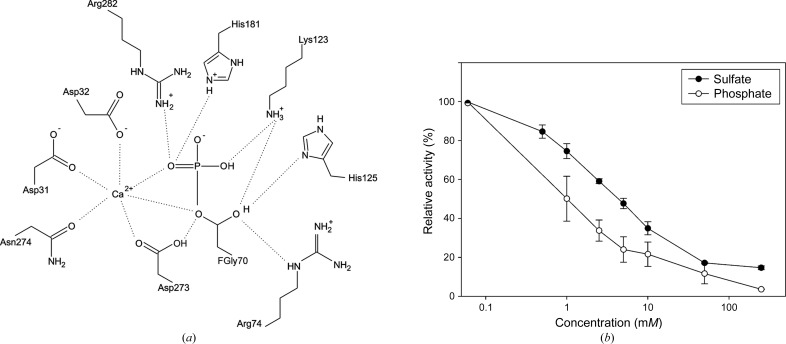
Active site and enzyme inhibition. (*a*) Schematic of the active-site region in SGSH. A Ca^2+^ ion is coordinated by side-chain P atoms from Asp31, Asp32, Asp273, Asn274 and the phosphorylated FGly70, which is in turn stabilized by interactions with the side chains of residues Arg74, Lys123, His125, His181, Asp273 and Arg282. (*b*) Inhibition of SGSH acitivity by phosphate and sulfate. The IC_50_ of phosphate was determined to be 1 m*M*; the IC_50_ of sulfate was 5 m*M*.

The consensus active site lies in domain 1 in a narrow pocket at the bottom of a surface cleft (Fig. 2 ▶ *c*) and close to the end of the first β-strand. Electron density consistent with the position of a divalent metal ion in O-sulfatases was interpreted as Ca^2+^ based on bond-valence calculations (Müller *et al.*, 2003 ▶). The metal ion is coordinated in a distorted, approximately octahedral arrangement by O atoms from the side chains of residues Asp31, Asp32, Asp273 and Asn274 and the phosphorylated FGly70, as shown in Fig. 2 ▶(*d*). A schematic overview of interactions in the active site is given in Fig. 3 ▶(*a*).

Since a clear differentiation between phosphate and sulfate as the species bound to FGly70 was not feasible based only on the electron density, we made quantitative measurements of the inhibitory effects of phosphate and sulfate on SGSH (Fig. 3 ▶ *b*). The IC_50_ values determined for phosphate and sulfate were 1 and 5 m*M*, respectively, indicating preferential phosphate binding. Since 20 m*M* K_2_HPO_4_ was present in the purification buffer, the electron density joined to FGly70 was modelled as a phosphate group. This crucial catalytic residue is stabilized by multiple interactions. The bridging O atom between C^β^ of FGly70 and the phosphate moiety coordinates Ca^2+^, while the hydroxyl O atom interacts with the side chains of Arg74, Lys123 and His125. The phosphate is further stabilized by interactions between its distal O atoms and Ca^2+^, and the side chains of Lys123, His181 and Arg282.

### 3.5. Homology of SGSH with O-sulfatases   

**Figure 4:**
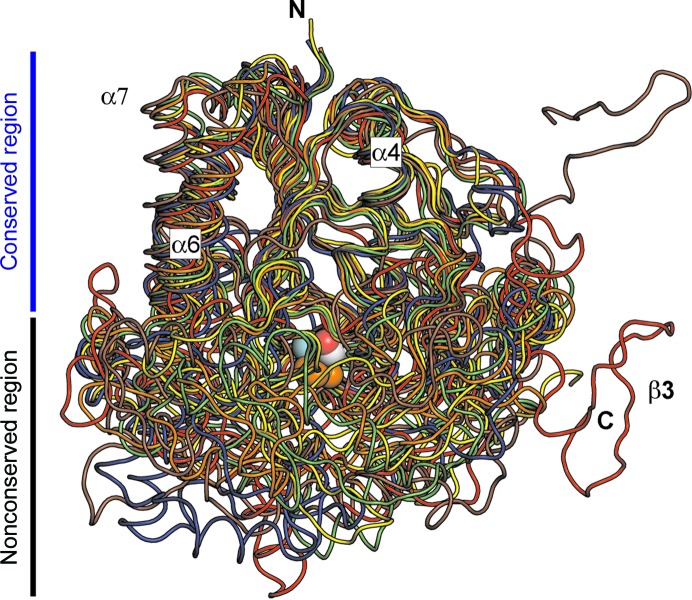
Superposition of the SGSH backbone on those of five related sulfatases: SGSH (red), ASA (orange), ASB (yellow), PAS (blue), GALNS (green) and BcPMH (brown). SGSH shares a common fold with O-sulfatases consisting of a large central β-sheet with decorating helices (‘conserved region’, top); the loops form a more variable region (‘nonconserved region’, bottom). For orientation, the SGSH N-terminus and C-terminus are shown (N and C, respectively), as are some secondary-structure elements (as in Fig. 2 ▶ *a*) and some atoms in the active site in ball representation: Ca^2+^ (dark grey), phosphate O atoms (orange), FGly C^β^ (light grey) and free hydroxyl O atom (red).

A Protein Data Bank (PDB) database search revealed only low sequence identities with known structures of O-sulfatases, ranging between 19 and 25% (Table 2 ▶). Five of these O-sulfatases catalyze the hydrolysis of an S—O bond in sulfate esters; the sixth is a phosphonate monoester hydrolase from *Burkholderia caryophylli* PG2952 (BcPMH) which acts on a broader range of substrates, including phosphate monoesters, diesters and triesters, phosphonate monoesters, sulfate monoesters and sulfonate monoesters (van Loo *et al.*, 2010 ▶). Fig. 4 ▶ shows an overlay of the SGSH main chain (red) with five of these closest O-sulfatase homologues. The SGSH structure shares with other N- and O-sulfatases the conserved fold of a large central β-sheet decorated by α-helices on both sides. Structural differences are smallest in this area, while the loops protruding from this region, and the C-terminal domain, display a significant variability among these sulfatases. The active site lies at the boundary of the two regions. The entry to the active site traverses the unconserved region, presumably reflecting the structural differences between the different substrates of these enzymes. Although the catalytic centres of these sulfatases show a high degree of conservation; there are a few exceptions (Table 2 ▶), including the replacement of Arg282 in SGSH by a lysine in the O-sulfatase homologues.

## 4. Discussion   

In the current study, we describe the first structure of sulfamidase, deficient activity of which causes the disease Sanfilippo A syndrome (also known as mucopolysaccharidosis IIIA). To our knowledge, it is also the first reported structure of a sulfatase that breaks an S—N sulfamate bond rather than an S—O sulfate-ester bond. The overall structure of the N-sulfatase SGSH subunit displays a characteristic sulfatase fold that is a member of the α/β-hydrolase fold family (Nardini & Dijkstra, 1999 ▶). The enzyme forms a homodimer in both of the two crystal forms for which structures were solved, with a calculated molecular weight of the mature unglycosylated subunit of 55 kDa. A comparison of all ten independent subunits in two different crystal forms of SGSH revealed low structural flexibility within the subunit. However, the dimer interface is slightly flexible, displaying a rotation of up to 3.0° in the two crystal forms.

### 4.1. Active site   

Residues neighbouring FGly70 in the active site are generally highly conserved between the N-sulfatase SGSH and the closest O-sulfatase homologues for which structures are available, excluding BcPMH. Notable exceptions are Arg282 (SGSH; discussed below), which is replaced by a lysine in these O-sulfatases, and the Ca^2+^-binding residue Asn274, which is replaced by a glutamine in ES. All ten important active-site residues in SGSH are conserved in the closest homologous sequences from diverse vertebrates and invertebrates that are tentatively annotated as SGSHs (Supplementary Fig. 1 ▶).

An *in silico* prediction was made that histidines are not involved in the active site of sulfamidase from *Flavobacterium heparinum*, thus distinguishing it from the active site of O-sulfatases (Myette *et al.*, 2009 ▶). In human SGSH, both histidines are involved in active-site interactions, as in O-sulfatases. However, a high degree of structural variability between SGSH and O-sulfatases begins in the immediate neighbourhood of the active-site residues, specifically in the short tunnel leading to the active site and its surrounding surface cleft. This presumably enables SGSH and O-sulfatases to accommodate distinct substrates that undergo a similar enzymatic reaction.

The crystallization conditions contained Mg^2+^ rather than Ca^2+^ ions. The latter ion is assumed to have bound to the enzyme intracellularly during expression and is the typical divalent cation for sulfatases (Table 2 ▶ and references therein). However, we are unable to exclude the possibility that at least some Mg^2+^ is also present in the metal-binding site.

### 4.2. Enzymatic reaction mechanism   

**Figure 5:**
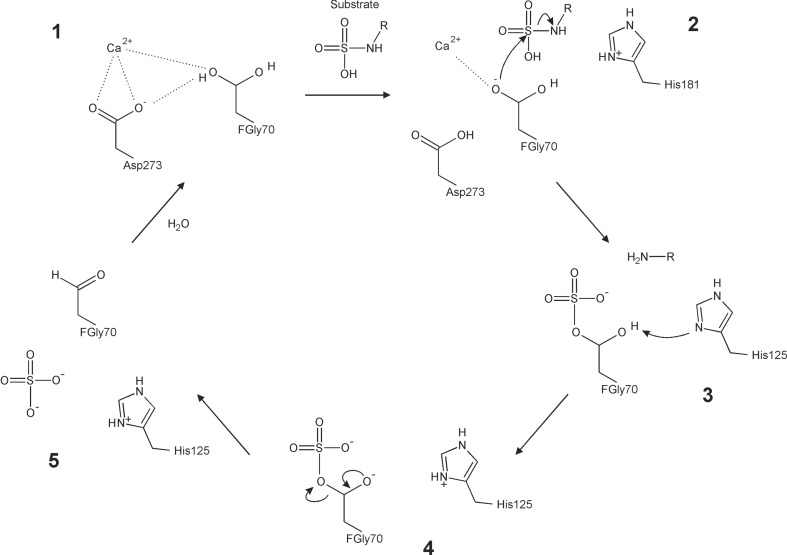
Proposed reaction mechanism in SGSH (schematic). The active-site formylglycine (FGly70), which is intrinsically reactive, undergoes hydration to form the resting state of the enzyme with a *gem*-diol group (step 1). Coordination of one of the hydroxyl groups of the *gem*-diol to a Ca^2+^ ion facilitates the development of a negative charge on the O atom as its proton is lost to a base. The negatively charged O atom nucleophilically attacks the sulfur centre of the N-linked sulfate group on the glucosamine substrate (step 2), resulting in a covalently bound enzyme–substrate complex with a pentavalent sulfur transition state. An acid (possibly His181) facilitates the cleavage of the S—N bond by protonating the bridging N atom to form an amine leaving group on the N-desulfated substrate, which diffuses away, leaving an O-sulfated enzyme (step 3). Finally, in a step that underlines the importance of the formylglycine residue, another base (His125) deprotonates the second hydroxyl group, resulting in a negatively charged O atom (step 4) that forms a double bond with the C^β^ atom as the C—O bond between it and the bridging O atom of the sulfate group breaks, eliminating the sulfate ion and regenerating the formylglycine residue (step 5).

SGSH catalyzes the cleavage of the S—N bond in *N*-sulfoglucos­amine, desulfating the glycos­aminoglycan substrates heparan sulfate and heparin at the non­reducing terminus of the linear GAG chain. In analogy with the enzymatic reaction mechanism previously proposed for O-sulfatases (Boltes *et al.*, 2001 ▶; von Bülow *et al.*, 2001 ▶), we suggest that the substrate is first desulfated while sulfating the enzyme, which is then desulfated in turn (Fig. 5 ▶). Specifically, an activated O atom, O^γ2^, from the hydrated form of formylglycine attacks the sulfur centre of the N-linked sulfate group of the substrate, resulting in a covalently bound enzyme–substrate complex with a pentavalent sulfur in the transition state. In this step, the activation of the hydroxyl O atom involves transfer of its proton to a base, with a potential candidate being Asp273. An acidic group then facilitates the breakage of the S—N bond by protonating the N atom to form an amine leaving group. The N-desulfated substrate diffuses away, leaving an O-sulfated enzyme. Finally, a base (possibly His125) deprotonates the second C^β^ hydroxyl group of formylglycine, resulting in the formation of a double bond between the O atom and the C^β^ atom. While the bridging C^β^—O bond to the sulfate group breaks, the sulfate ion is eliminated and the formylglycine residue is regenerated. The enzyme is now ready for another round of catalysis.

**Figure 6:**
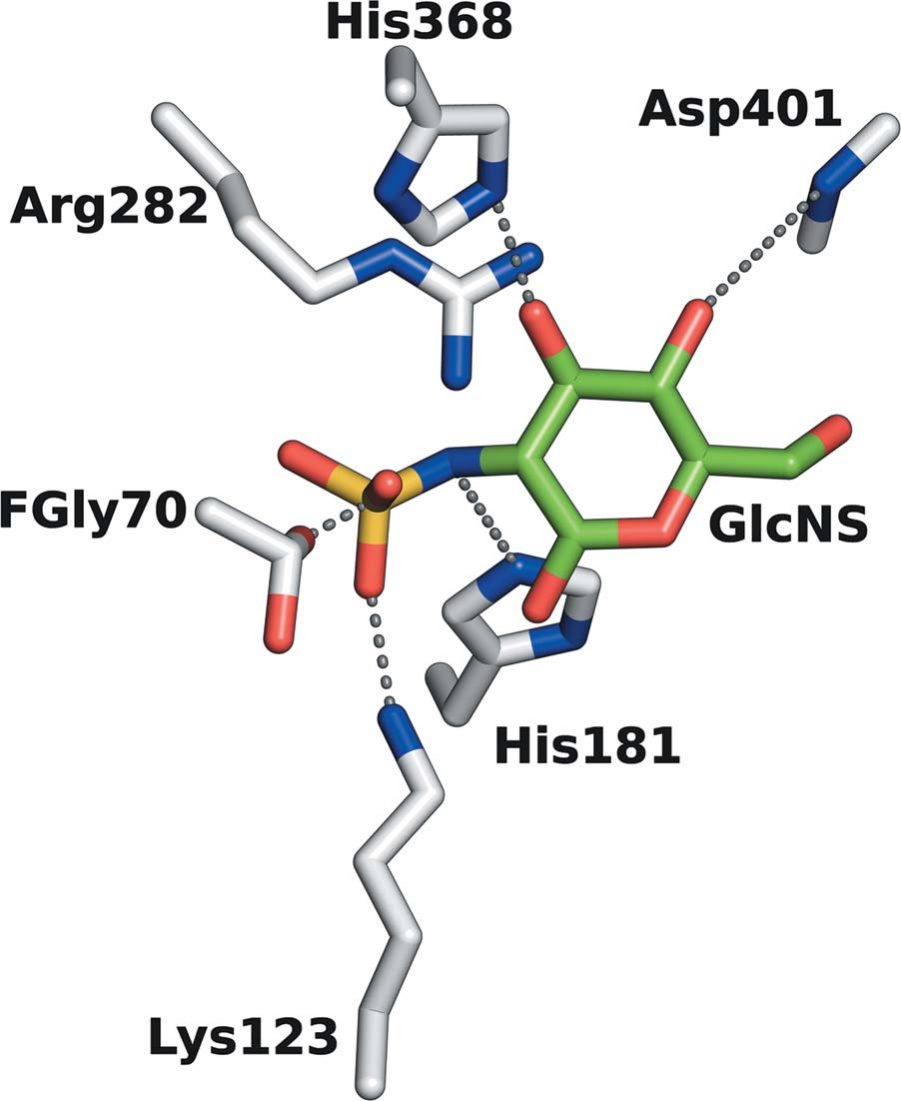
Hypothetical model showing some proposed interactions between the terminal *N*-sulfoglucosamine residue (GlcNS; C atoms in green, other atoms in standard colours) of the substrate with the enzyme in the active site (C atoms in light grey). His181 acts as the acid facilitating desulfation of the substrate. Other residues that help to bind and orient the substrate include the side chains of FGly70, Lys123, Arg282 and His368 and the main-chain amide N atom of Asp401.

The identity of the acid that facilitates the breakup of the transition state with concomitant desulfation of the substrate is uncertain, with candidates suggested for PAS including Lys375, His211, the second hydroxyl group of FGly *via* the sulfate, or a water molecule. The species that act as the acid could plausibly change as a function of solution pH (Boltes *et al.*, 2001 ▶). The role of His211 as the acid has also been suggested by a recent density functional theory-based quantum-mechanical study based on the PAS structure (PDB entry 1hdh; Marino *et al.*, 2013 ▶). The residue corresponding to His211 is conserved in the other sulfatases, including SGSH (His181; Table 2 ▶). In order to identify plausible interactions in the active site, we generated an *in silico* docking model of nonphosphorylated SGSH derived from the present study with the substrate monosaccharide 2-*N*-sulfoglucosamine (Fig. 6 ▶). In this model, His181 is located close to the N atom of the leaving amine, at the site of the cleaved S—N bond. His181 would be expected to be protonated at the lysosomal pH and thus appears to be a good potential candidate proton donor in the catalytic mechanism of SGSH.

The lysine equivalent to Lys375 in PAS is structurally conserved in all of these O-sulfatases, including BcPMH. However, in SGSH the lysine is replaced by an arginine (Arg282; SGSH numbering). The side chain of Arg282 forms salt bridges in the active site with Asp32 and Asp399, which are located 7.6 Å apart on opposite sides of this arginine (Fig. 2 ▶ *d*). An arginine at this position appears to be conserved in many putative sulfamidase sequences closely homologous to SGSH (Supplementary Fig. 1 ▶ 1). Furthermore, arginine has been shown to interact up to 2.5 times more strongly with heparin than does lysine (Stenlund *et al.*, 2002 ▶; Fromm *et al.*, 1995 ▶). In the docking model mentioned above, the side chain of Arg282 lies close to one of the sulfate O atoms and the 3-hydroxyl O atom of the substrate, apparently orienting the substrate for catalysis. Taken together, these structural data suggest an important binding role for Arg282 in SGSH. The functional and structural effects resulting from the substitution of arginine by lysine will be investigated using an Arg282Lys SGSH mutant in the future.

Since our structural data cannot completely describe the reaction partners involved, we are unable to exclude an alternative reaction mechanism in which one of the sulfate O atoms attacks the C^β^ atom (Bond *et al.*, 1997 ▶).

### 4.3. Enzyme inhibition   

Phosphate buffer was used in the purification of SGSH. Difference density next to FGly70 in the active site could be modelled as a covalently bound phosphoryl group. However, based on our data we are unable to exclude that this is a covalently bound sulfate group. Sulfate and phosphate have both been found to inhibit arylsulfatase A from rabbit liver (Lee & Van Etten, 1975 ▶) and human *N*-acetylgalactosamine-6-sulfatase (Bielicki *et al.*, 1995 ▶). Sulfate has previously been shown to be a strong inhibitor of SGSH (Freeman & Hopwood, 1986 ▶). We found phosphate to be a more potent inhibitor of SGSH than sulfate, which supports the building of a phosphorylated FGly70 in the present structure.

### 4.4. Molecular basis of disease-causing mutations   

The correlation of genotype with phenotype has often been difficult in MPS IIIA, with attendant difficulties in diagnosis and prognosis (Di Natale *et al.*, 1998 ▶; Yogalingam & Hopwood, 2001 ▶). Suggested explanations include genetic heterogeneity and difficulty in phenotypic classification, especially in a disease in which mental and behavioural symptoms typically dominate the clinical picture. Genetic, epigenetic and environmental modulating factors might further contribute to the phenotypic variability in MPS IIIA (Beesley *et al.*, 2000 ▶; Di Natale *et al.*, 1998 ▶; Perkins *et al.*, 1999 ▶). Except for two relatively small regions of high-homology sulfatase consensus-sequence regions (residues 70–80 and 115–124), SGSH in general shares only a low sequence identity with O-sulfatases for which atomic structures have been described. As a result, understanding the molecular basis of the disease has been especially difficult for this enzyme.

**Figure 7:**
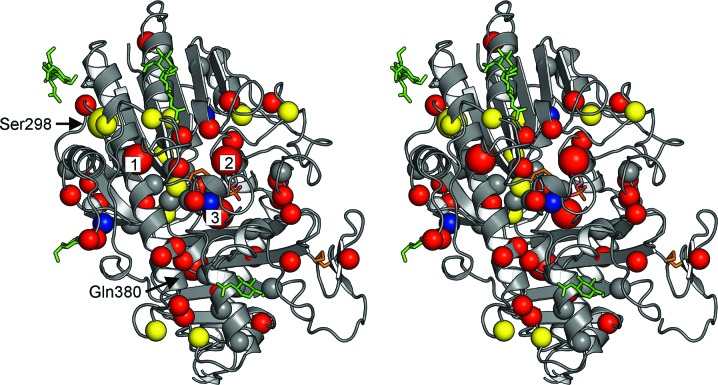
Stereo figure showing missense mutations mapped onto the structure of the SGSH monomer. C^α^ atoms of residues associated with an early-onset phenotype are shown in red, those associated with an intermediate-onset phenotype in blue and those associated with a late-onset phenotype in yellow. Missense mutations for which the phenotype was not reported are indicated in grey. Most mutations with known phenotype are early-onset mutations. Late-onset mutations appear to map closer to the periphery of the enzyme. Some of the most common mutations are indicated by a larger ball size. These are Ser298, Arg245 (indicated ‘1’), Arg74 (‘2’), Ser66 (‘3’) and Gln380. The orientation shown is the same as for one of the subunits (on the left) of the dimer in Fig. 2 ▶(*b*); the active site is indicated by FGly70 (stick model; standard colours) and Ca^2+^ ion (black ball). Glycosylations are shown as green sticks.

In Table 3 ▶, we list 80 disease-causing missense mutations that have been described in SGSH and briefly describe the predicted structural effects of these mutations based on the atomic structure of the enzyme determined in the present study. Fig. 7 ▶ displays these mutations as mapped onto the three-dimensional structure of the SGSH monomer. Most missense mutations for which disease severity has been reported in MPS IIIA patients are associated with a rapidly progressing, early-onset form of the disease (Di Natale *et al.*, 2003 ▶). Approximately a quarter of the mutations affect surface residues. In the case of two mutations that affect buried residues but are associated with a late-onset phenotype, namely E292K and S298P, it appears plausible that the presence of buried waters in the wild-type genotype acts as an ameliorating factor by offering substitution space for the mutated side chain.

Three of the mutations (D32G, D32E and D273N) affect buried Ca^2+^-binding residues. While D32G and D273N lead to an early-onset disease phenotype, the conservative substitution D32E is associated with late-onset disease, presumably owing to a partly retained Ca^2+^-binding capability.

#### 4.4.1. Common mutations   

Significant regional variations in the frequency of particular mutations have been described in MPS IIIA patients. Thus, R74C occurs in 56% of disease alleles in Polish patients, 50% of those in patients from Finland and Estonia and 21% of those in patients from Germany (Bunge *et al.*, 1997 ▶). Arg74 is identified in the present structure as a buried active-site residue that forms hydrogen bonds to FGly70 and salt bridges with the Ca^2+^-binding Asp31 and Asp273. A structural comparison with SGSH homologues with known atomic structures shows that it is strictly conserved in all of them, including BcPMH, which otherwise shows only a loose conservation of five of the ten important active-site residues (Table 2 ▶). Mutations affecting it would be predicted to impair enzyme function, likely including its stability, resulting in an early-onset disease phenotype, which has been observed for both mutations that affect this residue (R74C and R74H).

S66W is associated with an early-onset disease phenotype. It is the most common mutation observed in Italy, occurring in 33% of disease alleles (Di Natale *et al.*, 1998 ▶). Replacement of the buried Ser66 by the bulky aromatic residue tryptophan is likely to disrupt packing in a five-residue loop immediately preceding a helix (α2) housing active-site residues, including FGly70.

Another common mutation, R245H, is also associated with an early-onset phenotype and has been described in up to 57.8% of patients from the Netherlands, 35% of disease alleles from German patients and 20% of those from patients in the UK (Beesley *et al.*, 2000 ▶; Weber *et al.*, 1997 ▶; Bunge *et al.*, 1997 ▶). The arginine side chain is not typically buried. However, in SGSH the side chain of Arg245, which lies in the middle of the long helix α7, makes a buried salt bridge with Asp179 and hydrogen bonds to Asp179 and Phe197. A histidine in its place would be too bulky at the base and too short, thus tending to destabilize the local structure and packing. This interpretation is consistent with the lack of enzyme activity that has been reported for this mutation (Perkins *et al.*, 1999 ▶).

#### 4.4.2. Mutations and the dimer interface   

Active SGSH has been shown to be a dimer (Freeman & Hopwood, 1986 ▶; Paschke & Kresse, 1979 ▶). Ten chains of SGSH monomers found in the two crystal forms in the current study associate to form five homodimers. Residues forming the dimer interface were clearly identified. One of the missense mutations directly affects a residue at the dimer interface, namely V486F. Its replacement by the bulky aromatic phenylalanine would lead to a steric clash, destabilizing the dimer interface. The mutation has been shown to be associated with an early-onset disease phenotype.

Additionally, it has consistently been observed that nonsense mutations in the SGSH gene are associated with an early-onset disease phenotype (Yogalingam & Hopwood, 2001 ▶). The enzyme structure offers a rational molecular explanation. Many residues involved in dimer formation not only belong to the C-terminal domain 2 but also lie in close proximity to the C-terminus, including Leu487, Glu488, Pro497, Leu498 and the third-last residue Asn500. Nonsense mutations where even a relatively minor part of domain 2 is missing may be predicted to be destabilizing to the dimer interface. Since part of the access to the active-site pocket is formed by the second subunit in the dimer, this would be likely to affect the binding of heparin and heparan sulfatate and thus disrupt enzyme function.

### 4.5. Prospects   

The wild-type structure provides a rational basis for understanding the effects of many mutations. It may be useful in predicting the phenotype of mutations of unreported phenotype or as yet unknown genotype. Although it is possible to envisage significant divergence from the wild-type structure in some mutations, the low structural flexibility of SGSH suggests a promising effect of molecular chaperones in the cases of many missense mutations (Boyd *et al.*, 2013 ▶). Molecular chaperones that bind to the active site and reconstitute its structural architecture might be promising at first hand. In addition, small molecules with allosteric or stabilizing effect may be beneficial in the cases of mutations located more distantly from the active site or at the SGSH surface. *In vitro* studies that test for the rescue of SGSH activity may be the initial step to evaluate structure-based chaperones before clinical trials can further prove the effectiveness of these small molecules. Additionally, the SGSH structure will be very useful for the engineering of SGSH variants or fusion proteins with beneficial biological features that increase its therapeutic effectiveness in enzyme-replacement therapy and other treatment modalities (Sly & Vogler, 2013 ▶; Sorrentino *et al.*, 2013 ▶).

## 5. Conclusions   

The structure of SGSH determined to 2.0 Å resolution clearly extends our understanding of the molecular pathology underlying MPS IIIA and thus lays the groundwork for the development of the structure-based rational design of general and mutation-specific therapeutic tools such as molecular chaperones. In addition, the crystal structure provides fundamental atomic-level knowledge for protein modifications, with the aim of facilitating the transport of SGSH across the blood–brain barrier. Chemically synthesized protein modifications or genetically engineered fusion proteins can enter the brain *via* endogenous receptor-mediated endocytosis of the attached ligand (Pardridge, 2007 ▶). Since the brain is the organ most severely affected in MPS IIIA, cerebral delivery is absolutely crucial for successful treatment of this devastating disorder.

## Supplementary Material

PDB reference: sulfamidase, 4mhx

PDB reference: 4miv

Supporting Information.. DOI: 10.1107/S1399004714002739/cb5050sup1.pdf

## Figures and Tables

**Table 1:** Data-collection and refinement statistics The number of atoms is the occupancy sum; *B* factors are occupancy-weighted means. Solvent atoms were excluded when calculating the mean *B* factors for individual chains.

Crystal form	S	L
PDB code	4mhx	4miv
Space group	*P*2_1_	*P*2_1_
Unit-cell parameters		
*a* ()	61.4	103.0
*b* ()	107.9	211.6
*c* ()	79.8	108.4
()	104.1	102.7
Data collection		
Wavelength ()	0.99989	0.99989
Resolution range† ()	44.32.00 (2.102.00)	48.92.40 (2.502.40)
No. of reflections measured	452472	599199
No. of unique reflections	67940	174779
Multiplicity†	6.65 (6.38)	3.41 (3.45)
*R*_merge_ †	0.0886 (0.6539)	0.0702 (0.4723)
*R* †	0.0500 (0.5504)	0.0594 (0.4917)
Completeness† (%)	99.9 (99.8)	99.3 (98.7)
Mean *I*/(*I*)†	11.20 (1.85)	11.03 (2.08)
Refinement		
Resolution limit ()	44.32.00	48.852.40
*R*_free_ (No. of reflections)	0.2299 (3447)	0.2447 (8462)
*R*_work_	0.1920	0.2157
Total No. of reflections (working set)	64437	166273
Solvent content (%)	45.9	53.0
No. of molecules in the asymmetric unit	2	8
No. of non-H atoms refined	7968	31122
No. of water molecules refined	205	475
Mean *B* factors (^2^)		
Protein atoms	44.7	64.5
Backbone atoms	43.5	64.2
Side-chain atoms	45.9	64.8
Water atoms	39.0	37.6
Chain *A*	45.5	48.9
Chain *B*	43.9	44.1
Chain *C*		48.3
Chain *D*		44.9
Chain *E*		85.5
Chain *F*		75.8
Chain *G*		79.6
Chain *H*		93.0
R.m.s.d.s from ideal geometry		
Bond lengths ()	0.0125	0.0028
Bond angles ()	1.564	0.683
Ramachandran statistics		
Favored region (%)	97.1 (933/961)	97.2 (3727/3835)
Allowed region (%)	99.8 (959/961)	99.7 (3823/3835)
Outlier region (%)	0.21 (2/961)	0.31 (12/3835)

† Values in parentheses are for the outermost resolution shell.

**Table 2:** Structurally equivalent active-site residues classified by (putative) function in SGSH and closely homologous sulfatases with known atomic structures ASA, arylsulfatase A (also known as human lysosomal cerebroside-3-sulfate 3-sulfohydrolase; Lukatela *et al.*, 1998 ▶); ASB, arylsulfatase B (human lysosomal N-acetylgalactosamine-4-sulfate 4-sulfohydrolase (Bond *et al.*, 1997 ▶); PAS, arylsulfatase from *P. aeruginosa* (Boltes *et al.*, 2001 ▶); ES, human oestrone/dehydroepiandrosterone sulfatase (Hernandez-Guzman *et al.*, 2003 ▶); GALNS, human lysosomal (*N*-acetyl)galactosamine-6-sulfatase (Rivera-Coln *et al.*, 2012 ▶); BcPMH, sulfatase/hydrolase from *B. caryophylli* PG2952 (van Loo *et al.*, 2010 ▶). Sequence identities were calculated for protein sequences using the *PROMALS*3*D* server (Pei *et al.*, 2008 ▶) and C*lustalW*2 (BcPMH; Goujon *et al.*, 2010 ▶). R.m.s.d.s were calculated using *Coot*. Lys123 (SGSH numbering) and its equivalent residues in homologues also participate in sulfate binding.

Enzyme	SGSH	PAS	ASA	ASB	GALNS	ES	BcPMH
PDB code	4mhx	1hdh	1auk	1fsu	4fdi	1p49	2w8s
Sequence identity (%)	100	22.4	22.2	19.7	22.8	19.1	24.6
R.m.s.d. () (No. of residues)	0.00 (482)	2.17 (341)	2.21 (331)	2.18 (312)	1.97 (336)	1.95 (303)	1.98 (345)
Desulfation	FGly70	FGly51	FGly69	FGly91	FGly79	FGly75	FGly57
Metal	Ca^2+^	Ca^2+^	Mg^2+^ (Ca^2+^)†	Ca^2+^	Ca^2+^	Ca^2+^	Fe
Metal binding	Asp31	Asp13	Asp29	Asp53	Asp39	Asp35	Asp12
	Asp32	Asp14	Asp30	Asp54	Asp40	Asp36	
	Asp273	Asp317	Asp281	Asp300	Asp288	Asp342	Asp324
	Asn274	Asn318	Asn282	Asn301	Asn289	Gln343	His325
FGly binding	Arg74	Arg55	Arg73	Arg95	Arg83	Arg79	Arg61
	Lys123	Lys113	Lys123	Lys145	Lys140	Lys134	Tyr105
	His125	His115	His125	His147	His142	His136	Thr107
Sulfate binding	His181	His211	His229	His242	His236	His290	His218
	Arg282	Lys375	Lys302	Lys318	Lys310	Lys368	Lys337

† The identity of the divalent cation was later demonstrated to be Ca^2+^ in ASA structures with PDB codes 1n2k and 1n2l (Chruszcz *et al.*, 2003 ▶).

**Table 3:** Missense point mutations in SGSH and their expected effect based on the atomic structure of SGSH Abbreviations: NR, not reported; Interm., intermediate; sc, side chain, H bond, hydrogen bond; NASA, normalized accessible surface area per atom per residue (as a percentage of the maximal value for any internal residue in SGSH).

Protein	Codon	Phenotype	NASA	Type	Effect of mutation on structure	Reference
M1V	1A>G	NR		Signal peptide	Part of signal peptide	Pollard *et al.* (2013 ▶)
L12Q	35T>A	Late		Signal peptide	Part of signal peptide	Valstar *et al.* (2010 ▶)
A30P	88G>C	NR	0	Buried	Steric clash close to Ca^2+^-binding site; loss of H bond to Thr271	Pollard *et al.* (2013 ▶)
D32G	95A>G	Early	1	Metal binding	Disruption of Ca^2+^ binding	Beesley *et al.* (2000 ▶)
D32E	96C>A/G	Late	1	Metal binding	Altered Ca^2+^ binding	Meyer *et al.* (2008 ▶)
G33R	97G>A	NR	4	Buried	Introduces bulky sc next to Ca^2+^-binding Asp32	Pollard *et al.* (2013 ▶)
Y40N	118T>A	Interm.	13	Surface	Loss of H bonding to Leu294 and Phe60, and of -stacking interactions; next to glycosylation site	Di Natale *et al.* (1998 ▶)
N42K	126C>A	Early	6	Surface	Loss of H bonding to Ala44, Ile45 and Tyr240; steric clash	Lee-Chen *et al.* (2002 ▶)
A44T	130G>A	Early	31	Surface	Steric clash at surface site	Di Natale *et al.* (1998 ▶), Esposito *et al.* (2000 ▶)
S66W	197C>G	Early	2	Buried	Introduction of bulky sc in buried position in loop close to active site	Blanch *et al.* (1997 ▶), Weber *et al.* (1997 ▶), Di Natale *et al.* (1998 ▶), Montfort *et al.* (1998 ▶), Beesley *et al.* (2000 ▶), Chabs *et al.* (2001 ▶), Piotrowska *et al.* (2009 ▶), Valstar *et al.* (2010 ▶), Muschol *et al.* (2011 ▶), Pollard *et al.* (2013 ▶)
R74C	220C>T	Early	0	Buried	Disruption of ion pairs/H bonds with Ca^2+^-binding Asp31, FGly70 and Asp273; possible interference with disulfide-bridge formation	Bunge *et al.* (1997 ▶), Weber *et al.* (1997 ▶), Di Natale *et al.* (1998 ▶), Beesley *et al.* (2000 ▶), Esposito *et al.* (2000 ▶), Emre *et al.* (2002 ▶), Muschol *et al.* (2004 ▶, 2011 ▶), Meyer *et al.* (2008 ▶), Piotrowska *et al.* (2009 ▶), Valstar *et al.* (2010 ▶), Pollard *et al.* (2013 ▶)
R74H	221G>A	Early	0	Buried	Disruption of ion pairs/H bonds with Ca^2+^-binding Asp31, FGly70 and Asp273	Bunge *et al.* (1997 ▶), Chabs *et al.* (2001 ▶)
T79P	235A>C	Early	0	Buried	Disruption of H bonding to Ala75, Ser76 and Leu81	Weber *et al.* (1997 ▶), Beesley *et al.* (2000 ▶), Pollard *et al.* (2013 ▶)
H84Y	250C>T	Early	1	Buried	Loss of H bond to Ser364 and Thr475; steric clash in buried position	Beesley *et al.* (2000 ▶)
Q85R	254A>G	Early	3	Buried	Steric clash	Montfort *et al.* (1998 ▶), Chabs *et al.* (2001 ▶)
M88T	263T>C	NR	0	Buried	Destabilizes van der Waals interactions in buried position; steric clash	Fiorentino *et al.* (2006 ▶)
G90R	268G>A	Early	0	Buried	Gain of bulky sc in buried position; change of / angles	Bunge *et al.* (1997 ▶), Piotrowska *et al.* (2009 ▶)
S106R	318C>A	Late	0	Buried	Loss of H bond to Leu109, Val131; clash possibly accommodated within longer, partially surface-exposed loop	Muschol *et al.* (2004 ▶)
T118P	352A>C	NR	0	Buried	Loss of H bonds to Asp135; destabilizes -sheet	Zhang Huiping (2008 ▶)
G122R	364G>A	Interm.	1	Buried	Bulky sc in buried position; Gly / angles	Bunge *et al.* (1997 ▶), Di Natale *et al.* (1998 ▶), Beesley *et al.* (2000 ▶), Valstar *et al.* (2010 ▶), Pollard *et al.* (2013 ▶)
P128L	383C>T	Late	22	Surface	Favourably surface-exposed to minimize steric clash; in loop with FGly-binding His125 and Lys123	Di Natale *et al.* (1998 ▶, 2003 ▶)
V131M	391G>A	Early	1	Buried	Bulky sc in buried position; destabilizes loop with FGly-binding residues	Weber *et al.* (1997 ▶)
T139M	416C>T	Early	1	Buried	Bulky sc in buried position; loss of H bond to Glu141	Weber *et al.* (1997 ▶)
L146P	437T>C	Early	11	Surface	Loss of H bond to Ser144; some clash at surface; destabilizes helix 5; close to glycosylation site (Asn151)	Di Natale *et al.* (1998 ▶)
R150W	448C>T	Early	1	Buried	Introduction of bulky aromatic sc; loss of salt bridge with Asp179, H bonding to His181	Beesley *et al.* (2000 ▶), Chabs *et al.* (2001 ▶)
R150Q	449G>A	Early	1	Buried	Loss of ion pair with Asp179, H bonding to His181; next to glycosylated Asn151	Bunge *et al.* (1997 ▶), Di Natale *et al.* (1998 ▶), Chabs *et al.* (2001 ▶), Valstar *et al.* (2010 ▶), Montfort *et al.* (1998 ▶)
L163P	488T>C	Early	8	Buried	Disruption of hydrophobic interactions, H bond to Val159; clash; destabilizes helix 6	Muschol *et al.* (2004 ▶)
D179N	535G>A	Early	1	Buried	Loss of buried salt bridges with Arg150, Arg245	Di Natale *et al.* (1998 ▶), Esposito *et al.* (2000 ▶)
P180L	539C>T	Late	0	Buried	Some steric clash next to active site-residues Asp31 and His181	Valstar *et al.* (2010 ▶)
R182C	544C>T	Interm.	4	Buried	Loss of ion pair with Asp235, H bond to Pro277 close to active site; possible interference with disulfide-bridge formation	Di Natale *et al.* (1998 ▶), Valstar *et al.* (2010 ▶)
G191R	571G>A	Early	11	Surface	Surface-exposed but steric clash with scs of Glu195 and Lys196; Gly / angles	Muschol *et al.* (2004 ▶), Valstar *et al.* (2010 ▶)
F193L	579C>G	NR	0	Buried	Disrupts -stacking next to active-site loop (His181)	Bunge *et al.* (1997 ▶), Yogalingam Hopwood (2001 ▶)
R206P	617G>C	Late	73	Surface	Suface-exposed; Arg206 has no backbone amide H bond to lose; close to glycosylated Asn151; change in / angles	Montfort *et al.* (1998 ▶), Esposito *et al.* (2000 ▶), Chabs *et al.* (2001 ▶), Gabrielli *et al.* (2005 ▶)
P227R	680C>G	Early	0	Buried	Steric clash from bulky substitution disrupts packing in buried position	Di Natale *et al.* (1998 ▶), Esposito *et al.* (2000 ▶)
A234G	701C>G	Early	51	Surface	Unclear; possibly destabilization of helix 7	Weber *et al.* (1997 ▶)
D235N	703G>A	Early	1	Buried	Loss of buried salt bridge with Arg182 and of H-bond acceptor	Beesley *et al.* (2000 ▶), Lee-Chen *et al.* (2002 ▶)
D235V	704A>T	NR	1	Buried	Loss of buried salt bridge with Arg182 and of H bonds to Thr192 and Thr407	Bunge *et al.* (1997 ▶)
T242T	726C>T	NR	0	Buried	Unclear	Valstar *et al.* (2010 ▶)
R245H	734G>A	Early	0	Buried	Loss of buried salt bridge with Asp179 and H bonds to Asp179 and Cys194; clash; packing of helix 7	Blanch *et al.* (1997 ▶), Bunge *et al.* (1997 ▶), Weber *et al.* (1997 ▶, 1998 ▶), Beesley *et al.* (2000 ▶), Muschol *et al.* (2004 ▶, 2011 ▶), Meyer *et al.* (2008 ▶), Valstar *et al.* (2010 ▶), Pollard *et al.* (2013 ▶)
D247H	739G>C	NR	1	Buried	Loss of H bonding to Leu50; clash	Valstar *et al.* (2010 ▶)
G251A	752G>C	Late	13	Surface	Some clash with sc of His49 in surface-exposed site	Meyer *et al.* (2008 ▶), Muschol *et al.* (2011 ▶)
D273N	817G>A	Early	2	Metal binding	Disrupts Ca^2+^ binding	Beesley *et al.* (2000 ▶)
Y286S	857A>C	NR	4	Buried	Disruption of H bond to Glu437 and of -stacking interactions	Yogalingam Hopwood (2001 ▶)
P288S	862C>T	Early	5	Buried	Possibly unsatisfied H bonding in sc of Ser in buried position	Emre *et al.* (2002 ▶)
P288L	863C>T	NR	5	Buried	Steric clash	Pollard *et al.* (2013 ▶)
E292K	874G>A	Late	0	Buried	Buried water might offer space to accommodate larger sc	Piotrowska *et al.* (2009 ▶)
P293T	877C>A	NR	3	Buried	Steric clash; loss of Pro from three-residue loop	Di Natale *et al.* (2006 ▶)
P293S	877C>T	Early	3	Buried	Unclear; loss of Pro from three-residue loop	Lee-Chen *et al.* (2002 ▶), Pollard *et al.* (2013 ▶)
S298P	892T>C	Late	1	Buried	Loss of H bonds to Glu300, His301, but steric clash milder as buried water offers substitution space; favourable / angles (Ser297, Ser298)	Bunge *et al.* (1997 ▶), Beesley *et al.* (2000 ▶), Muschol *et al.* (2004 ▶, 2011 ▶), Meyer *et al.* (2008 ▶), Valstar *et al.* (2010 ▶), Pollard *et al.* (2013 ▶)
E300V	899A>T	Early	48	Surface	Unclear; loss of surface salt bridge with Arg23; little steric clash	Bekri *et al.* (2005 ▶)
R304L	911G>T	NR	7	Surface	Loss of surface salt bridge with Glu355 and of H bonds to Ala351 and Gln307; some steric clash	Di Natale *et al.* (2006 ▶), Pollard *et al.* (2013 ▶)
Q307P	920A>C	Early	38	Surface	Loss of surface H bonds to Arg304; steric clash; destabilization of strand 8	Bekri *et al.* (2005 ▶)
A311D	932C>A	NR	3	Buried	Steric clash; buried charge; unsatisfied H bonding	Pollard *et al.* (2013 ▶)
D317H	949G>C	NR	4	Buried	Steric clash; loss of H bonds to Ser314, Arg346	Pollard *et al.* (2013 ▶)
T321A	961A>G	NR	0	Buried	Loss of H bonds to Asp317, Leu348 and of van der Waals interactions	Bunge *et al.* (1997 ▶)
I322S	965T>G	Late	0	Buried	Loss of van der Waals interactions, but I322S can H-bond to Leu318	Beesley *et al.* (2000 ▶)
S347Y	1040C>A	NR	15	Surface	Bulky aromatic in solvent-exposed position, but with minimal steric clash; loss of H bonds to Asp324, Leu349	Valstar *et al.* (2010 ▶)
S347F	1040C>T	Late	15	Surface	Bulky aromatic in solvent-exposed position, but with minimal steric clash; loss of H bonds to Asp324, Leu349	Miyazaki *et al.* (2002 ▶)
A354P	1060G>C	Early	58	Surface	Loss of H bond to Pro350; steric clash with Pro350; change in / angles	Montfort *et al.* (1998 ▶), Chabs *et al.* (2001 ▶)
E355K	1063G>A	Early	39	Surface	Loss of surface salt bridge with Arg304 and of H bonds to Ser309, Glu310; charge switch	Beesley *et al.* (2000 ▶)
S364R	1092C>G	NR	1	Buried	Loss of H bonds to Gln83 and His84; marked steric clash in buried position	Bunge *et al.* (1997 ▶)
E369K	1105G>A	Early	6	Surface	Loss of H bond to Gln400; charge switch close to active site	Di Natale *et al.* (1998 ▶, 2003 ▶), Esposito *et al.* (2000 ▶), Valstar *et al.* (2010 ▶), Pollard *et al.* (2013 ▶)
Y374H	1120T>C	Early	2	Buried	Unsatisfied H bonding; charge	Beesley *et al.* (2000 ▶)
R377C	1129C>T	Early	0	Buried	Loss of buried salt bridge with Asp477 and of H bonds to Ser366, Met376; plausibly interference with disulfide-bridge formation	Di Natale *et al.* (1998 ▶), Lee-Chen *et al.* (2002 ▶)
R377H	1130G>A	Early	0	Buried	Loss of buried salt bridge with Asp477 and of H bonds to Ser366, Met376	Weber *et al.* (1997 ▶), Yogalingam Hopwood (2001 ▶), Bunge *et al.* (1997 ▶), Valstar *et al.* (2010 ▶)
R377L	1130G>T	NR	0	Buried	Loss of buried salt bridge with Asp477 and of H bonds to Ser366, Met376	Pollard *et al.* (2013 ▶)
Q380R	1139A>G	Early	2	Buried	Gain of charge in buried position close to surface; steric clash may affect H bond to Arg382	Weber *et al.* (1997 ▶), Valstar *et al.* (2010 ▶)
L386R	1157T>G	Early	0	Buried	Introduction of charge and steric clash in buried position; disruption of hydrophobic interactions	Montfort *et al.* (1998 ▶), Chabs *et al.* (2001 ▶)
V387M	1159G>A	NR	2	Buried	Bulky residue in buried position	Di Natale *et al.* (2006 ▶)
N389S	1166A>G	NR	0	Buried	Loss of buried H bonds to Ala434, Glu437	Pollard *et al.* (2013 ▶)
N389K	1167C>A	NR	0	Buried	Loss of buried H bonds to Ala434, Glu437; steric clash	Bunge *et al.* (1997 ▶), Valstar *et al.* (2010 ▶)
L411R	1232T>G	NR	1	Buried	Introduction of charge in buried position; steric clash; disruption of hydrophobic interactions	Valstar *et al.* (2010 ▶)
T415P	1243A>C	NR	34	Surface	Loss of H bond to Leu411; steric clash with Leu411; kink in helix 11 close to glycosylation site	Pollard *et al.* (2013 ▶)
T421R	1262C>G	Late	16	Surface	Loss of H bond to Trp423; solvent exposure accommodates bulky sc	Valstar *et al.* (2010 ▶)
R433W	1297C>T	Early	6	Buried	Loss of buried H bonds to Asn284, Tyr430 and of charge; steric clash	Beesley *et al.* (2000 ▶), Yogalingam Hopwood (2001 ▶), Chabs *et al.* (2001 ▶), Muschol *et al.* (2004 ▶), Pollard *et al.* (2013 ▶)
R433Q	1298G>A	Early	6	Buried	Loss of buried H bonds to Asn284, Tyr430 and of charge; destabilizes packing	Chabs *et al.* (2001 ▶), Di Natale *et al.* (2003 ▶), Valstar *et al.* (2010 ▶)
D444G	1331A>G	Late	13	Surface	Loss of surface H bonds to Thr448, Gln449	Miyazaki *et al.* (2002 ▶)
E447K	1339G>A	Early	8	Surface	Switch of charge in partly buried location	Blanch *et al.* (1997 ▶), Chabs *et al.* (2001 ▶)
Q472H	1416G>C	NR	6	Surface	Loss of H bond to Asp477	Pollard *et al.* (2013 ▶)
V486F	1456G>T	Late	37	Dimer interface	Disruption of dimer interface	Beesley *et al.* (2000 ▶)

## Footnotes

1 Supporting information has been deposited in the IUCr electronic archive (Reference: CB5050).
