# Supplementary material for: Structure of sulfamidase provides insight into the molecular pathology of mucopolysaccharidosis IIIA
Source: Acta Crystallogr D Biol Crystallogr. 2014 Apr 30;70(Pt 5):1321–35. doi: 10.1107/S1399004714002739 (PMC4014121; doi:10.1107/S1399004714002739)
Supplement: Supplementary file 1 [file d-70-01321-sup1.pdf]

Acta Crystallographica Section D

Volume 70 (2014)

Supporting information for article:

Structure of sulfamidase provides insight into the molecular pathology of mucopolysaccharidosis IIIA

Navdeep S. Sidhu, Kathrin Schreiber, Kevin Pröpper, Stefan Becker, Isabel Usón, George M. Sheldrick, Jutta Gärtner, Ralph Krätzner and Robert Steinfeld

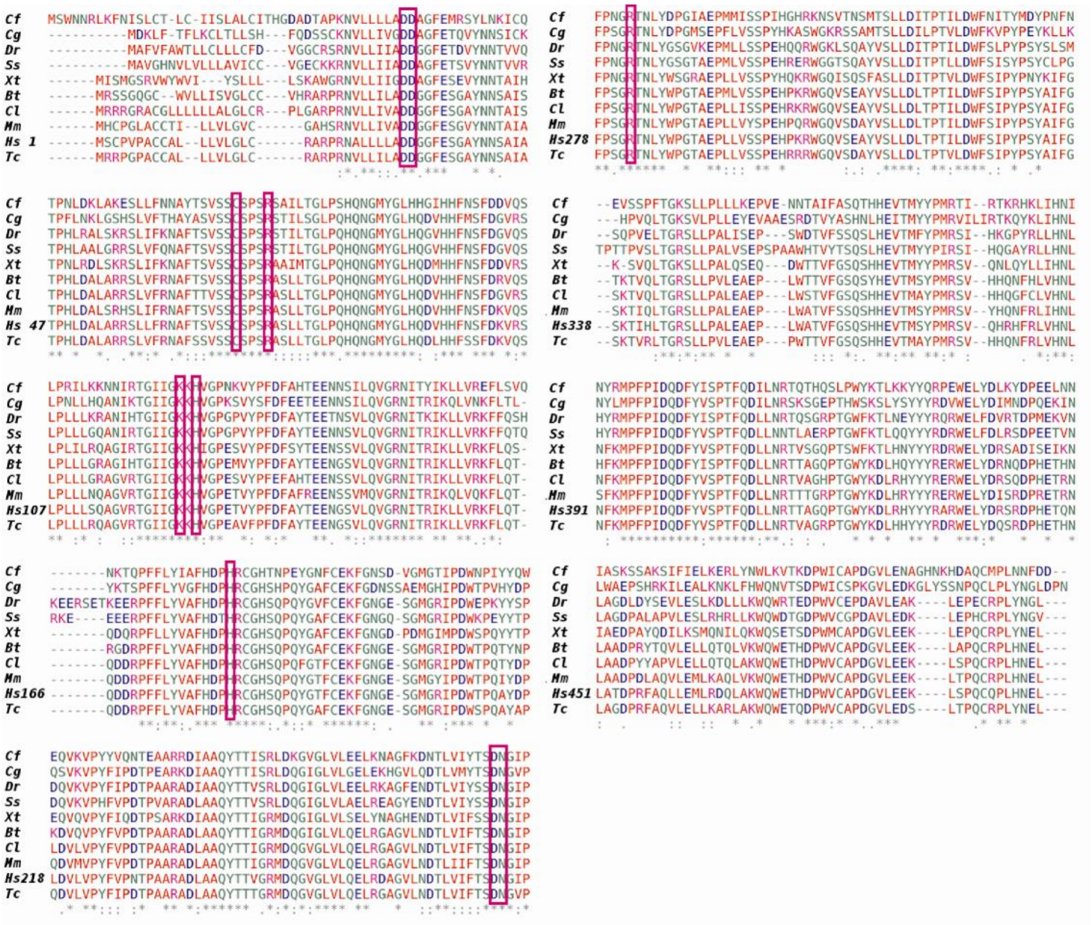

Figure S1 Multiple sequence alignment of human SGSH and homologous sequences. All 10 important active site residues are highlighted. Numbering shown is for human SGSH. Sequences homologous to human SGSH were retrieved using the BLASTP server of the National Library of Medicine. Closest sequences annotated as SGSH were blindly selected from the results and sequence alignment performed using CLUSTAL Omega. Abbreviations: Cf, *Camponotus floridanus* (Florida carpenter ant), EFN70845.1; Cg, *Crassostrea gigas* (Pacific oyster), EKC39134.1; Dr, *Danio rerio* (zebrafish), NP_001116740.1; Ss, *Salmo salar* (Atlantic salmon), NP_001133784.1; Xt, *Xenopus tropicalis* (western clawed frog), NP_001120065.1; Bt, *Bos taurus* (domestic cow), NP_001095659.2; Cl, *Canis lupus familiaris* (dog), NP_001003114.1; Mm, *Mus musculus* (house mouse), AAF29460.1; Hs, *Homo sapiens*, NP_000190.1; Tc, *Tupaia chinensis* (Chinese tree shrew), ELW68227.1.

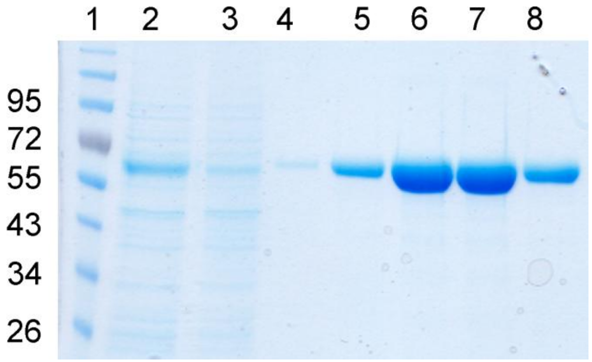

Figure S2 SDS-PAGE image showing several steps of SGSH purification. Lane 1: Protein size markers, molecular weights are indicated at the left side in kDa. Lane 2: crude supernatant from cell culture containing recombinantly expressed SGSH. Lane 3: Flow through. Lane 4 and 5: Fractions eluted from the column at 100 mM imidazole. Lane 6 to 8: Fractions eluted from the column at 250 mM imidazole that were used for crystallisation trials.
